# Length-Weight Relationship and Condition Factor of Fishes in Two Major Rivers, the Chao Phraya and the Bang Pakong, in Thailand

**DOI:** 10.21315/tlsr2024.35.1.14

**Published:** 2024-03-30

**Authors:** Chayajit Deekrachang, Chaiwut Grudpun, Apinun Suvarnaraksha, Pisit Phomikong, Tuantong Jutagate

**Affiliations:** 1 Sustainable Fisheries Research Center, Faculty of Agriculture, Ubon Ratchathani University, 85 Sathonlamak Rd, Mueang Si Khai, Warin Chamrap District, Ubon Ratchathani 34190, Thailand; 2 Faculty of Fisheries Technology and Aquatic Resources, Maejo University, Nong Han, San Sai District, Chiang Mai 50290, Thailand; 3 Freshwater Fisheries Research and Development Division, Department of Fisheries, 50 Phothonyothin Rd., Lat Yao, Chatuchak, Bangkok 10900, Thailand

**Keywords:** Chao Phraya River, Bang Pakong River, Fish Stock, Well-Being, Body Shape

## Abstract

Length-weight relationship (LWR) was used as a tool to assess the status of fish stocks, through condition factor, in major rivers in Thailand. Fifty-one fish species from each river, i.e., The examined for LWR using 11 years of monitoring data (2010–2020) for Bang Pakong and 14 years of monitoring data (2007–2020) for Chao Phraya, which comprised 57,871 samples. The parameters for LWR and condition factor were examined by species and by body shape, i.e., ovate, oblong, elongate or eel-like. The coefficient of determination (*r^2^*) of all log-transformed LWRs was greater than 0.90. Parameter *b* of LWR ranged between 2.06 and 3.46 (median = 3.00) for fishes from the Chao Phraya River and between 1.72 and 3.68 (median = 2.76) for fishes from the Bang Pakong River. The overall condition factor, which implies the well-being that indicates the health or fattening of the fishes in a stock, ranged between 0.93 and 1.09. There was no significant difference in the overall well-being (*P =* 0.279) between the two rivers. Fishes with oblong and elongate shapes in the Chao Phraya River showed higher median values of parameter *b* of LWR than those from the Bang Pakong River. However, there was no significant difference (*P >* 0.05) in the well-being of the fish stocks between the two rivers when pooled by shape. The findings are fundamental information for fish stock assessment in the two rivers, which greatly support the small-scale fisheries in Thailand.

**Highlights**

- Providing, for the first time, length-weigh relationships (LWR) of 51 fish species in two major rivers of Thailand, using long-term data series, i.e., 9 years.
- The median values of parameter *b* of LWR were 3.00 and 2.76 for the Chao Phraya and the Bang Pakong rivers, respectively, implying isometric and negative allometric growth for most of the fish species in the respective rivers.
- Condition factor of the studied species was compared between the two rivers, both by individual species and by shape, and indicated that most of the fishes were in good condition of well-being.

## INTRODUCTION

Thailand hosts a great diversity of freshwater fishes, where 828 species, including 13 elasmobranch fishes are recorded in FishBase (Froese & Pauly 2022). The number of indigenous species is as high as 661, of which 2.3% are endemic (Nguyen & De Silva 2006). This exceptional fish diversity, incorporated with an extensive inland water area (about 4.5 × 10^4^ km^2^) within seven major river basins, supports and contributes to about 7% of the country’s overall fish production (Jutagate *et al*. 2016). Inland fisheries in Thailand are small in scale; the catch was estimated as roughly 116,000 metric tonnes in 2020 (Fisheries Development Policy and Planning Division 2022). Management of inland fisheries in Thailand is done more scientifically in lakes and reservoirs, due to the more reliable data for fish landings and hence greater certainty of stock status, compared to rivers and neighbouring inundated areas (Charernnate *et al*. 2021). Nevertheless, data from regular fish sampling in the rivers by the Inland Fisheries Research and Development Division, Department of Fisheries, which is commonly used to monitor the status of fish diversity and variation in catch per unit effort (e.g., Saenghong *et al*. 2021; Noonin *et al*., can be also applied to examine the biometric information of individual fish stocks, such as their length-weight relationship (LWR).

The LWR is a fundamental tool in fisheries science. A synthetic analysis of LWR for a species can provide insightful understanding into the biology, ecology and physiology of that species (Froese 2006; Sánchez-González *et al*. 2020). The LWR is used for estimating the weight corresponding to a given length, and also implies the well-being of individual fish: the heavier the fish of a given length, the better the well-being of the fish (Froese 2006; Al Nahdi *et al*. 2016). This relationship is commonly further incorporated in the analyses of fish population dynamics and stock assessment, as well as in modelling the ecosystem. In these analyses, the weight in each length class of an individual species is used to estimate that stock’s biomass (Hilborn & Walters 1992; Kulbicki *et al*. 2005; Froese 2006; Al Nahdi *et al*. 2016). The LWR can be explained by the power function of length as predictor and weight as response as well as parameters *a* and *b*. For LWR, parameter *b* describes the curve of the relationship, and generally differs among species and stocks (Pope & Kruse 2007). This parameter also indicates whether the growth of fish is allometric or isometric (Froese 2006; Damchoo *et al*. 2021). Meanwhile, parameter *a* is a scaling coefficient for the weight at length of the fish species (Kuriakose 2017). This parameter is applied with parameter *b* to estimate weight, and the two values are negatively correlated; a large value of *b* is associated with a small value of *a*, and vice versa (Froese 2006).

The well-being, i.e., condition or fatness, of fish in the stock can be examined by the condition factor, which is derived from LWR. The condition factor can used to assess many facets of fish populations, for example, health of fish stock, and the effects of environmental conditions and management measures (Pope & Kruse 2007). Although there are many equations for calculating condition factor, Le Cren’s (1951) relative condition factor (K_rel_) is recommended when exploring relative condition of individuals within a sample (Froese 2006). The K_rel_ is estimated by comparing the observed weight of an individual with the mean weight for that length, i.e., predicted by LWR; the fish is assumed to be fit when K_rel_ is equal to 1 (Le Cren 1951; Froese 2006, Jisr *et al*. 2018). The K_rel_ is also a useful indicator to monitor fish stress of a population, based on the species-specific LWR across a broader geographical range, which allows comparison of well-being across populations (Swingle & Shell 1971; Pope & Kruse 2007).

The previous studies in LWR of fishes in Thailand were mainly incorporated with fish stock assessment for marine fishes but few for freshwater fishes (e.g., Wongyai *et al*. 2020; Charernnate *et al*. 2021; Damchoo *et al*. 2021). To our knowledge, there are neither studies on condition factor nor stock status of fishes inhabiting any rivers in Thailand, which are required as the crucial information for appropriate fisheries management. The main objective of this study is, therefore, to provide fundamental biometric information, which could be further used for enhancing fisheries management and conservation. In this study, we investigated the LWR and condition factor, by K_rel_, of fishes, as individual species and pooled by body shape, in the two major rivers in Thailand, namely the Chaophrya and the Bang Pakong, which are considered as the country’s main fishing grounds for river fisheries.

## MATERIALS AND METHODS

### Studied Rivers, Sampling Sites and Data Collection

**Figure 1:**
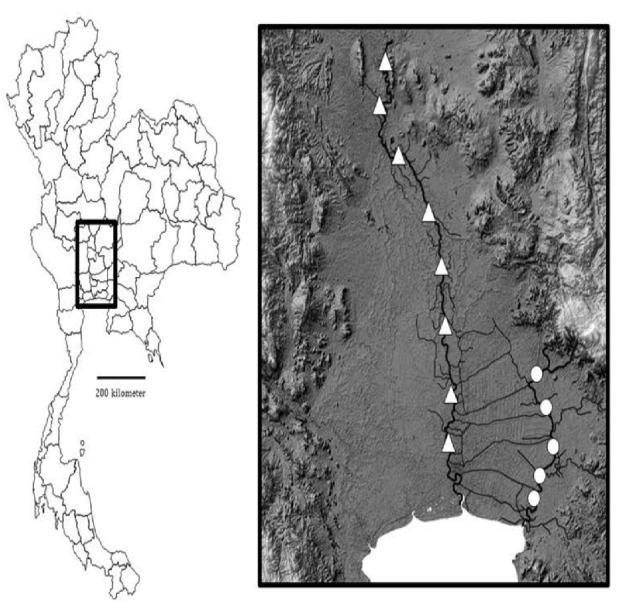
Map of Thailand and fish sampling stations along the Chao Phraya (triangles) and Bang Pakong (circles) rivers.

The Chao Phraya River (CPR, Fig. 1) lies within the central region of Thailand, with a basin area of 160,000 km^2^. The river originates in Nakhon Sawan Province, where it is formed from the confluence of the Ping and Nan rivers. The CPR then runs southward for 372 km to enter the Gulf of Thailand at Bangkok (Avakul *et al*. 2022). According to FishBase (Froese & Pauly 2022), 328 fish species have been recorded in the CPR. The Bang Pakong River (BPR, Fig. 1) is a major river in eastern Thailand; it is 231 km long with a basin area of 17,900 km^2^ and runs to the Gulf of Thailand at Chachoengsao Province (Damchoo *et al*. 2021). Sawangarreruks *et al*. (2003) reported that 270 fish species were found in the BPR. Both rivers are under tropical climate, in which the dry and wet seasons are March to May and September to November, respectively. The two rivers are also influenced by the Southeast and Northeast monsoons, and seawater intrusion can reach as far as 100 km between March and April (Damchoo *et al*. 2021; Avakul *et al*. 2022).

Fish sampling sites were fixed and located along the two rivers and are marked for CPR (triangles) and BPR (dots) in Fig. 1. Fish monitoring was conducted by the Inland Fisheries Research and Development Division, Department of Fisheries, four times annually, to represent the dry and wet seasons as well as the transition periods between the two seasons. In each sampling event, three sets of gillnets with a series of mesh sizes (20 mm, 30 mm, 40 mm, 55 mm, 70 mm and 90 mm) were operated once for 12 h during nighttime, i.e., 6:00 p.m.-6.00 a.m. (Noonin *et al*. 2022). Fish specimens were identified to species in situ according to Nelson *et al*. (2016) and then measured for total length to the nearest 0.1 cm and weighed to the nearest 0.1 g (Froese 2006). The monitoring data from CPR (2010–2020) and BPR (2007–2020) was used in this study.

### Data Analysis

The length-weight relationship (LWR) was in the power function (Equation 1) and analysed by log-transformed regression as in Equation 2.


(1) $$W=aL^{b}$$

(2) $${log}_{10}W={log}_{10}a+b{log}_{10}L$$

where *W* is weight in grams, *L* is total length in cm, and *a* and *b* are parameters. Analysis of covariance (ANCOVA) was used to determine whether *log_10_a* and parameter *b* for the same species were different between the two rivers (i.e., with river as factor). Meanwhile, Le Cren’s (1951) relative condition factor, K_rel_, (Equation 3) was applied to examine the well-being, i.e., fatness, of individual specimens.


(3) $$K_{rel}=\frac{W}{aL^{b}}$$

**Figure 2:**
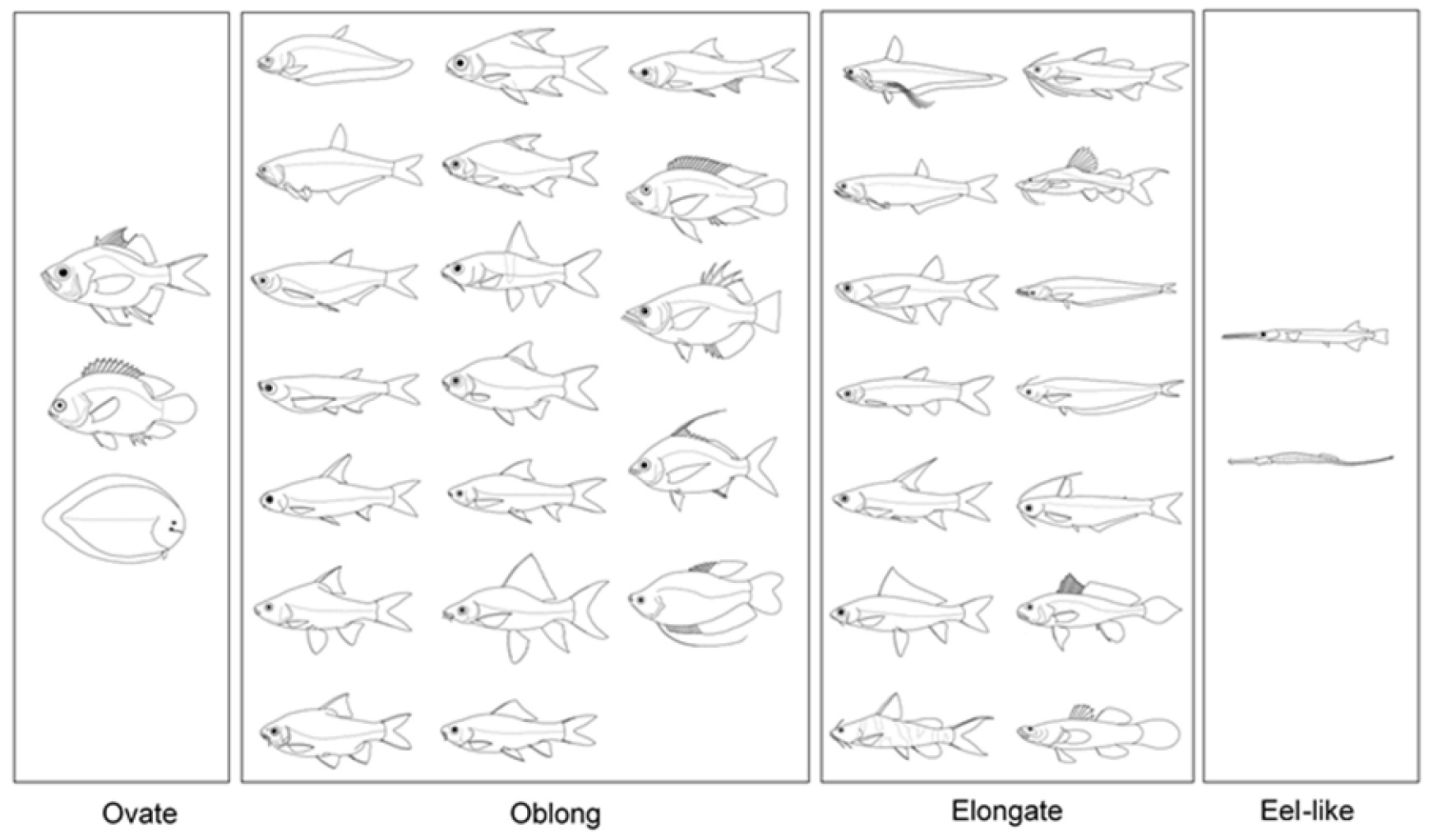
Representative fish species from 38 genera sampled from the Chao Phraya and Bang Pakong rivers, grouped by body shape.

The student’s t-test was used to determine any differences in well-being of the same species or body shape group (Fig. 2) between the two rivers. Moreover, variance of parameter *b* for each shape was calculated and differences between the two rivers were determined by F-test. All statistical analyses were carried out by using R version 4.2 (R Core team 2021).

## RESULTS

There were 57,871 individual fish specimens from 51 species, used for this study due to large sample sizes (all ≥ 30 individuals) (Table 1). The species were from 17 families, with Family Cyprinidae the most common. In terms of body shape, the studied species were mostly oblong (26 species), followed by elongate (18 species), ovate (5 species) and eel-like (2 species). The size range of the overall specimens was varied. The largest individual specimen by length was 50.8 cm, found in *Labeo chrysophekadion*. Meanwhile, the smallest specimen was 1.8 cm in *Parambassis siamensis*. These species also had the maximum and minimum individual weights, of 1,520.2 g found in *L. chrysophekadion*, and 0.2 g in *P. siamensis*. All of the studied species are targeted by fishing, and most are of “least concern” as classified in the IUCN Red List; however, five species were “data deficient,” namely *Boesemania microlepis*, *Doryichthys boaja*, *Hemibagrus filamentus*, *Kryptopterus cheveyi* and *Setipinna melanochir*.

**Figure 3:**
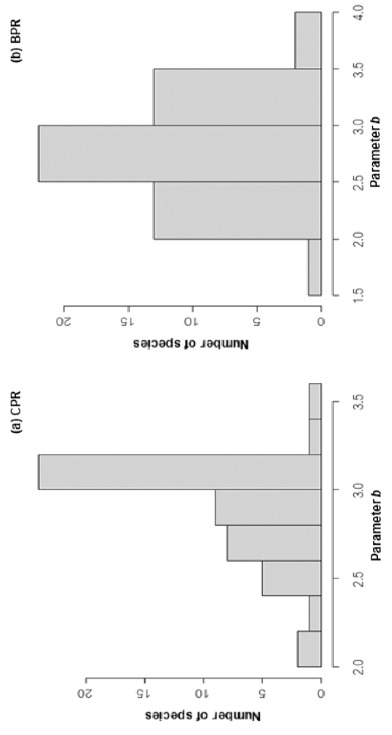
Histogram of length-weight relationship parameter *b* for fishes in (a) Chao Phraya River (CPR) and (b) Bang Pakong River (BPR).

Results from the log-transformed length-weight relationship (LWR), i.e., log_10_*a* and *b*, as well as relative condition factor (K_rel_) are shown in Table 2. The coefficient of determination (*r^2^*) of all studied species in each river exceeded 0.90, implying high correlation between length and weight, and showing that length is a good predictor of weight with high degree of accuracy. The value of log_10_*a* did not show significant difference (*P > 0.05*) either across species or between rivers. Parameter *b* ranged between 2.06 and 3.46 for fishes from the Chao Phraya River (CPR) and it was between 1.72 and 3.68 for fishes from the Bang Pakong River (BPR). The median values for parameter *b* were 3.00 for CPR and 2.76 for BPR; the latter value implies negative allometry, i.e., low fatness or the rate of increase in body length of the fish species is not proportional to the rate of increase in their body weight, for most of the fish species in BPR (Fig. 3). Out of 51 species, 17 showed no significant difference (*P* > 0.05) in parameter *b* between CPR and BPR, but all differed in *log_10_a*. The overall K_rel_ values ranged between 0.93 and 1.09. There was no significant difference in the overall well-being (*P =* 0.279) between CPR (mean K_rel_ ± SD = 1.03 ± 0.04) and BPR (1.04 ± 0.07). Ten species showed significant difference in K_rel_ (*P <* 0.05). *Labeo chrysophekadion*, *Xenentodon canciloides Pseudomystus siamensis* and *Oxyeleotris marmorata* were healthier in CPR than BPR, meanwhile *Notopterus notopterus*, *Cyclocheilos enoplos*, *Labiobarbus siamensis*, *Paralaubuca riveroi*, *Mystus mysticetus* and *Doryichthys boaja* showed higher K_rel_ in BPR than CPR.

**Figure 4:**
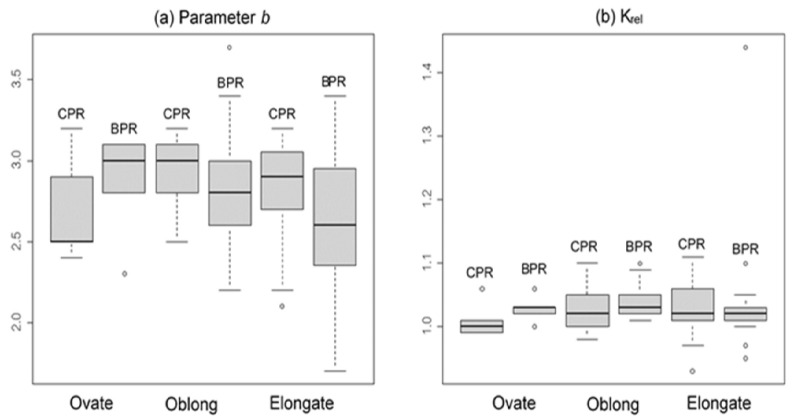
Boxplots of length weight relationship parameter *b* and relative condition factor, K_rel_, for fishes pooled by body shape from the Chao Phraya and Bang Pakong rivers.

Because there were only two eel-like (very elongate) species in the dataset, they were excluded for the analysis by body shape. Higher median values of parameter *b* were observed in CPR fishes with oblong or elongate shapes compared to BPR, however the value was higher in BPR than CPR for the fishes in ovate shape (Fig. 4). The F-test results indicated that the variances of parameter *b* between the two rivers were equal for elongate and ovate shapes, but higher in BPR for oblong fishes (*P* = 0.012). No significant differences (*P >* 0.05) were found in well-being between CPR and BPR fishes of all three body shapes (Fig. 4).

## DISCUSSION

The length-weight relationship (LWR), which had long been ignored by most of fisheries scientists, has been more recognised in the last decade because of the need for reliable body weight estimates for determining biomass and stock status (Froese 2006; Gerritsen & McGrath 2007; Froese *et al*. 2014). Moreover, only LWR parameters *per se* can reveal the robustness of the fish population (Gerritsen & McGrath 2007; Kuriakose 2017). This understanding is quite important for the river fishes, which are now undergoing a number of anthropogenic pressures, not only to fishes themselves but also their habitats, placing their populations at risk of collapse (Collen *et al*. 2014). Although almost all of the species from this study are of “least concern” in the IUCN Red List, their importance to fisheries justify the monitoring and assessment of their stock status. In this study, LWR parameters for 51 fish species were reported from two major Thai rivers. Our results, therefore, can be added to the LWR dataset in FishBase for better estimation of species-specific LWR parameters (Froese *et al*. 2014).

The number of individuals collected for our study conformed to the minimum sample size required for LWR analysis (Tessier *et al*. 2016; Sánchez- González *et al*. 2020). Although beyond the capacity of our data, variation in the obtained parameter *b*, although can be caused by many factors, but it is widely accepted that fishing intensity and food availability are main factors, which makes fishes from different stock grow in different rates (Haberle *et al*. 2023). Moreover, variation in *b* can be caused by the condition of individuals sampled, for example sex and maturity stage, as well as sampling and preservation methods (Jisr *et al*. 2018; Wongyai *et al*. 2020; Rahman *et al*. 2021). Tessier *et al*. (2016) studied LWR of eight fishes from a reservoir in Lao PDR, of which four species, namely *Cyclocheilichthys apogon*, *C. armatus*, *Puntius brevis* and *Parambassis siamensis*, were also analysed in our study. Interestingly, all four reservoir-sampled species presented higher values for *b* than those in our two studied rivers. The lower median value (i.e., including all species) of parameter *b* in the Bang Pakong River (BPR) suggests that most fishes in this system have a relatively slow growth rate and tend to be thinner when they get old (Froese 2006; Kuriakose 2017). This is a precaution to resource management since it likely shows the slow resilience of the population (Le Bris *et al*. 2015). The parameter *b* is theoretically near 3 and ranges between 2.5 and 3.5, since the weight of a 3-dimensional object is roughly proportional to the cube of length (Froese 2006; Kuriakose 2017; Jisr *et al*. 2018). The few cases in which parameter *b* was outside this common range, i.e., *Kryptopterus geminus* in CPR, *K. geminus* and *Xenentodon canciloides* in BPR, could be due to the limited size range of the samples (Froese 2006). It is worth noting that these three species indeed have atypical body shapes, which would cause their parameter *b* values to lie outside the common range (Al Nahdi *et al*. 2016). Moreover, Froese (2006) showed that among the body shapes, there is increasing fluctuation in LWR parameters as body length increases. Froese *et al*. (2014) also stated that systematic differences in *log_10_a* and *b* between body shapes are largely effects of different body plans.

Fluctuation in relative condition factor (K_rel_) values, as with LWR parameters, are normally due to fishing intensity and environmental stress, food availability, and condition of the fish (Le Cren 1951; Jisr *et al*. 2018). As the obtained K_rel_ fluctuated around 1, it can be concluded that our studied fish species in both rivers were in good growth condition and that the habitat was suitable, implying substantial carrying capacity to maintain fish stocks (Froese 2006; Jisr *et al*. 2018; Rahman *et al*. 2021; Haberle *et al*. 2023). Only one exotic species, *Oreochromis niloticus*, was collected in substantial number for our LWR and K_rel_ analyses. However, as Thailand is a paradise of many exotic fishes (Collen *et al*. 2014), the abundance and condition of these exotic species should be monitored, since their invasions may reduce the condition factor of native fishes in the same habitat (Iron *et al*. 2007). Water temperature is also considered to affect K_rel_ in temperate fish species since habitat temperature controls food consumption, growth rate and various body functions (Sabbir *et al*. 2020; Rahman *et al*. 2021). However, the low intra-annual variation in water temperature (range of 28°C–32°C) in both CPR (Avakul *et al*. 2022) and BPR (Saithong *et al*. 2022) is expected to have less effect on K_rel_.

## CONCLUSION

The LWR and K_rel_ reported for fishes in this study provides the baseline condition of many fisheries-targeted species in two major rivers of Thailand. The LWR results showed that most of fishes in the Chao Phraya River tended to be isometric growth, meanwhile most of the fishes in the Bang Pakong River were negative allometry. The relative condition factor of fishes in both rivers fluctuated around 1, implying suitability of environments. These findings can also facilitate the estimation of fish biomass from the regularly collected length frequency data, and the assessment of stock status of the studied fishes, which are both important for fisheries resource management. Moreover, our obtained LWR parameters can be added to the FishBase dataset for comparing the condition of these stocks to the same species elsewhere. Our study also supports the campaign of International Year of Artisanal Fisheries and Aquaculture (IYAFA 2022) of UN-FAO to strengthen the science-policy interface to sustain inland fisheries.

## Figures and Tables

**Table 1:** Family, species, sample size, range of weight, range of length and IUCN status of fishes of the Chao Phraya and Bang Pakong rivers, Thailand.

Family	Species	N	Length (cm)		Weight (g)		IUCN status

			L_min_	L_max_	W_min_	W_max_	
Notopteridae	*Notopterus notopterus*	383	7.0	34.0	2.0	351.6	LC
	*Coilia lindmani*	1,308	5.0	21.4	0.9	45.0	LC
Engraulidae	*Lycothrissa crocodilus*	316	6.1	29.1	1.4	147.2	LC
	*Setipinna melanochir*	915	4.8	25.6	0.7	114.8	DD
	*Paralaubuca barroni*	1,102	5.5	20.1	1.6	63.6	LC
	*Paralaubuca harmandi*	167	5.1	41.4	1.5	645.0	LC
	*Paralaubuca riveroi*	96	8.1	25.5	3.9	112.9	LC
	*Paralaubuca typus*	840	4.0	30.0	1.0	233.7	LC
	*Parachela siamensis*	3,309	4.0	16.0	0.4	30.1	LC
	*Esomus metallicus*	1,385	4.2	13.0	1.2	15.4	LC
	*Rasbora dusonensis*	1,657	4.0	15.0	0.6	22.4	LC
	*Amblyrhynchichthys micracanthus*	3,907	5.0	31.1	0.9	464.6	LC
	*Cyclocheilos enoplos*	1,083	4.6	48.2	1.6	1149.3	LC
	*Cyclocheilichthys apogon*	172	5.2	16.7	1.4	52.1	LC
	*Cyclocheilichthys armatus*	3,809	2.0	20.8	0.4	110.8	LC
	*Mystacoleucus obtussirostris*	1,764	3.8	20.5	1.0	55.9	LC
Cyprinidae	*Puntioplites proctozysron*	4,016	3.4	30.5	1.0	461.0	LC
	*Barbonymus altus*	7,596	3.0	36.0	0.5	632.8	LC
	*Barbonymus gonionotus*	3,710	3.3	40.0	1.6	806.5	LC
	*Barbonymus schwanenfeldi*	1,034	3.5	28.9	1.2	361.9	LC
	*Hampala macrolepidota*	464	3.0	39.9	2.3	739.0	LC
	*Puntius brevis*	656	4.5	11.8	0.9	18.6	LC
	*Gymnostomus siamensis*	2,132	3.3	25.0	1.8	213.8	LC
	*Labeo chrysophekadion*	245	6.0	50.8	3.6	1520.2	LC
	*Labiobarbus siamensis*	3,323	3.0	25.5	1.7	217.6	LC
	*Osteochilus vittatus*	475	4.0	21.8	1.9	141.6	LC
	*Thynnichthys thynnoides*	1,036	4.5	25.0	1.2	197.8	LC
	*Pseudomystus siamensis*	159	6.0	17.0	2.0	44.0	LC
	*Mystus mysticetus*	556	5.3	24.5	1.9	100.4	LC
Bagridae	*Mystus multiradiatus*	338	4.0	17.0	1.5	48.9	LC
	*Mystus singaringan*	519	6.5	26.6	1.4	98.7	LC
	*Hemibagrus filamentus*	300	6.5	39.9	2.6	521.7	DD
	*Hemibagrus spilopterus*	254	8.5	36.0	3.4	495.1	LC
	*Phalacronotus bleekeri*	224	5.4	50.0	2.1	700.0	LC
Siluridae	*Kryptopterus cheveyi*	566	4.0	23.0	1.0	42.5	DD
	*Kryptopterus geminus*	416	6.0	18.5	2.4	36.6	LC
Schibeidae	*Laides longibarbis*	1,292	6.0	26.0	1.5	106.1	LC
Belonidae	*Xenentodon canciloides*	163	9.6	38.5	4.0	80.2	LC
Syngnathidae	*Doryichthys boaja*	123	16.2	33.0	1.5	17.8	DD
Sciaenidae	*Boesemania microlepis*	239	5.0	50.7	1.1	913.7	DD
	*Parambassis siamensis*	850	1.8	7.5	0.2	5.9	LC
Ambassidae	*Parambassis wolffii*	2,844	2.7	18.8	0.2	114.7	LC
	*Parambassis apogonoides*	522	3.3	15.1	0.4	56.9	LC
Cichlidae	*Oreochromis niloticus*	90	7.4	35.6	6.2	770.0	LC
Toxotidae	*Toxotes chatareus*	150	5.3	21.3	2.0	184.8	LC
Gerreidae	*Gerres filamentosus*	85	4.9	18.9	1.8	88.6	LC
Butidae	*Oxyeleotris marmorata*	324	6.4	35.5	2.3	595.4	LC
Pristolepididae	*Pristolepis fasciata*	327	4.3	24.4	1.6	417.6	LC
Soleidae	*Brachirus panoides*	154	5.2	24.3	1.6	205.9	LC

Osphronemidae	*Trichopodus microlepis*	350	4.2	16.0	2.0	44.7	LC
	*Trichopodus trichopterus*	126	4.4	12.6	1.9	24.0	LC

*Note:* LC = least concern and DD = data deficient.

**Table 2:** Length-weight relationship parameters *log_10_a* and *b* as well as relative condition factor, K_rel_ (and SD), of individual fish species from the Chao Phraya and Bang Pakong rivers. A *P*-value less than 0.05 indicates statistically significant of each parameter between the two rivers.

Family	Species	Morphometry	Chao Phraya River				Bang Pakong River				*P*-value		

			*b*	log_10_*a*	K_rel_	SD	*b*	log_10_*a*	K_rel_	SD	*b*	log *a*	K_rel_
Notopteridae	*Notopterus notopterus*	Oblong	3.03	−5.17	0.98	0.17	3.16	−5.51	1.05	0.16	3.6E 02	2.2E 16	0.01324
Engraulidae	*Coilia lindmani*	Elongate	2.67	−4.72	1.02	0.17	2.37	−4.02	1.03	0.23	8.2E 01	2.0E 16	0.20441
	*Lycothrissa crocodilus*	Elongate	3.16	5.66	1.11	0.17	3.05	−5.33	0.97	0.21	1.9E 01	2.0E 16	0.59371
	*Setipinna melanochir*	Oblong	3.18	5.69	1.06	0.30	2.74	4.60	1.05	0.32	6.7E 11	2.2E 16	0.53064
Cyprinidae	*Paralaubuca barroni*	Oblong	2.72	4.60	1.02	0.17	2.92	−4.95	1.03	0.15	1.5E 02	2.2E 16	0.13691
	*Paralaubuca harmandi*	Oblong	3.05	−5.22	1.02	0.25	2.76	4.60	1.03	0.20	1.3E 01	2.0E 16	0.88851
	*Paralaubuca riveroi*	Oblong	3.10	−5.31	1.00	0.24	2.94	−5.02	1.07	0.16	4.6E 01	2.0E 16	0.00579
	*Paralaubuca typus*	Oblong	3.02	5.16	1.01	0.23	2.63	−4.32	1.03	0.23	3.2E 09	2.2E 16	0.35797
	*Parachela siamensis*	Oblong	2.68	−4.47	1.00	0.18	2.38	−3.88	1.01	0.20	1.4E 14	2.2E 16	0.48264
	*Esomus metallicus*	Elongate	2.20	−3.49	1.01	0.11	2.18	−3.47	1.01	0.10	6.3E 01	2.0E 16	0.37965
	*Rasbora dusonensis*	Elongate	2.26	3.63	1.01	0.10	2.21	−3.52	1.01	0.12	1.2E 01	2.0E 16	0.58858
	*Amblyrhynchichthys micracanthus*	Oblong	3.12	−5.23	1.01	0.25	2.49	−3.83	1.10	0.54	2.2E 16	2.2E 16	0.38028
	*Cyclocheilos enoplos*	Elongate	3.02	5.06	0.97	0.19	3.20	−5.50	1.10	0.30	1.3E 05	2.2E 16	0.00046
	*Cyclocheilichthys apogon*	Oblong	2.95	−4.90	0.98	0.12	3.04	−5.04	1.02	0.19	4.7E 01	2.2E 16	0.41794
	*Cyclocheilichthys armatus*	Oblong	2.89	4.76	1.05	0.36	2.76	−4.50	1.05	0.31	1.6E 03	2.2E 16	0.64060
	*Mystacoleucus obtussirostris*	Oblong	2.59	4.16	1.02	0.20	2.21	−3.41	1.03	0.36	8.3E 10	2.2E 16	0.28404
	*Puntioplites proctozysron*	Oblong	3.01	−4.88	1.03	0.25	3.03	−4.93	1.02	0.38	7.6E 01	2.0E 16	0.33797
	*Barbonymus altus*	Oblong	3.04	−4.94	0.99	0.27	3.05	4.96	1.04	0.46	4.5E 01	2.0E 16	0.15411
	*Barbonymus gonionotus*	Oblong	3.00	−4.88	1.03	0.21	2.66	−4.09	1.06	0.40	2.2E 16	2.2E 16	0.30895
	*Barbonymus schwanenfeldi*	Oblong	3.06	−4.99	1.04	0.28	2.68	−4.17	1.05	0.36	2.4E 11	2.2E 16	0.48428
	*Hampala macrolepidota*	Oblong	2.96	−4.84	1.05	0.23	2.58	−4.02	1.05	0.36	4.5E 10	2.2E 16	0.37369
	*Puntius brevis*	Oblong	2.80	−4.52	1.02	0.14	2.82	−4.55	1.01	0.16	7.6E 01	2.0E 16	0.11518
	*Gymnostomus siamensis*	Oblong	3.16	−5.30	1.01	0.21	2.65	−4.23	1.02	0.24	2.0E 16	2.0E 16	0.08437
	*Labeo chrysophekadion*	Oblong	3.05	−5.08	1.06	0.17	2.89	4.65	1.02	0.16	4.9E 03	2.2E 16	0.01075
	*Labiobarbus siamensis*	Elongate	3.16	−5.34	0.93	0.18	2.70	−4.37	1.05	0.42	2.0E 16	2.0E 16	0.00025
	*Osteochilus vittatus*	Oblong	3.03	−5.04	1.07	0.48	3.68	6.37	1.09	0.23	2.2E 16	2.2E 16	0.91053
	*Thynnichthys thynnoides*	Oblong	3.18	5.36	1.10	0.27	2.64	−4.21	1.03	0.25	2.0E 16	2.0E 16	0.16346
Bagridae	*Pseudomystus siamensis*	Elongate	3.03	−5.07	1.07	0.19	2.56	−4.10	1.01	0.17	4.0E 03	2.2E 16	0.01181
	*Mystus mysticetus*	Elongate	2.71	−4.44	1.02	0.19	3.37	−5.84	1.44	0.20	9.3E 14	2.2E 16	0.00002
	*Mystus multiradiatus*	Elongate	3.06	−5.20	1.07	0.21	2.25	3.64	1.02	0.22	6.1E 13	2.2E 16	0.30762
	*Mystus singaringan*	Elongate	2.91	−5.00	1.03	0.21	2.58	−4.24	1.03	0.21	2.3E 05	2.2E 16	0.47182
	*Hemibagrus filamentus*	Elongate	2.91	−4.90	0.99	0.25	2.83	−4.74	1.02	0.18	2.6E 01	2.0E 16	0.31711
	*Hemibagrus spilopterus*	Elongate	3.24	5.67	1.10	0.19	2.84	−4.77	1.00	0.15	2.6E 05	2.2E 16	0.22627
Siluridae	*Phalacronotus bleekeri*	Elongate	2.65	−4.55	1.06	0.33	2.40	−4.02	1.02	0.20	8.5E 02	2.0E 16	0.67206
	*Kryptopterus cheveyi*	Elongate	2.06	−3.32	1.01	0.12	2.28	−3.80	1.03	0.24	9.8E 03	2.2E 16	0.95525
	*Kryptopterus geminus*	Elongate	2.80	−4.80	1.01	0.18	1.72	2.63	1.01	0.15	2.2E 16	2.2E 16	0.49905
Schibeidae	*Laides longibarbis*	Elongate	2.80	−4.79	1.03	0.19	2.39	−3.84	1.02	0.20	2.2E 16	2.2E-16	0.76098
Belonidae	*Xenentodon canciloides*	Eel-like	3.10	−5.94	1.17	0.11	2.03	−3.43	1.02	0.21	1.9E 07	2.2E 16	0.00018
Syngnathidae	*Doryichthys boaja*	Eel-like	3.46	−7.47	1.09	0.11	3.53	7.62	1.22	0.12	6.9E 01	2.2E 16	0.00236
Sciaenidae	*Boesemania microlepis*	Elongate	3.06	−5.28	1.06	0.17	2.92	−4.89	1.03	0.50	9.1E 02	2.2E 16	0.07780
Ambassidae	*Parambassis siamensis*	Ovate	2.52	−4.03	0.99	0.15	2.26	−3.57	1.00	0.09	4.0E 06	2.2E 16	0.63803
	*Parambassis wolffii*	Ovate	2.88	−4.58	1.06	0.36	3.01	−4.84	1.06	0.26	2.1E 05	2.2E 16	0.53236
	*Parambassis apogonoides*	Ovate	2.46	−3.92	1.01	0.14	2.81	4.46	1.02	0.25	5.5E 06	2.2E 16	0.68887
Cichlidae	*Oreochromis niloticus*	Oblong	3.14	−5.07	1.08	0.24	3.05	−4.82	1.02	0.17	5.0E 01	2.0E 16	0.27174
Toxotidae	*Toxotes chatareus*	Oblong	3.06	−4.83	1.00	0.15	2.92	−4.58	1.02	0.18	1.2E 01	2.2E 16	0.17378
Gerreidae	*Gerres filamentosus*	Oblong	2.77	−4.32	1.00	0.14	3.35	−5.49	1.07	0.22	1.9E 04	2.2E 16	0.05959
Butidae	*Oxyeleotris marmorata*	Elongate	2.95	−4.78	1.01	0.17	3.22	−5.43	0.95	0.16	7.5E 06	2.2E 16	0.02034
Pristolepididae	*Pristolepis fasciata*	Ovate	3.18	−5.01	1.00	0.25	3.09	−4.89	1.03	0.28	8.5E 02	2.0E 16	0.90091
Soleidae	*Brachirus panoides*	Ovate	2.45	−3.73	0.99	0.28	3.07	−5.11	1.03	0.34	3.3E 04	2.2E 16	0.25250
Osphronemidae	*Trichopodus microlepis*	Oblong	2.70	−4.31	1.03	0.19	2.39	3.76	1.01	0.15	2.5E 04	2.2E 16	0.97957
	*Trichopodus trichopterus*	Oblong	2.51	−3.91	1.02	0.23	2.99	−4.83	1.01	0.16	2.9E 02	2.0E 16	0.75050
